# Trends in cancer mortality among the elderly in China, 2005–2035

**DOI:** 10.1371/journal.pone.0302903

**Published:** 2024-05-29

**Authors:** Meiyan Wang, Xunli Su, Yunhua Hu, Jian Yang

**Affiliations:** 1 Shihezi University School of Medicine, Shihezi, Xinjiang, China; 2 The First Affiliated Hospital, School of Medicine, Shihezi University, Shihezi, China; Debre Tabor University, ETHIOPIA

## Abstract

**Background:**

With the appearance and aggravation of the aging society, cancer has become one of the major problems that threaten the life and health of Chinese residents seriously.

**Objective:**

To explore the cancer epidemiological characteristics among the elderly in China from 2005 to 2016, and to provide strategies for cancer prevention and treatment.

**Methods:**

Stratified analysis was conducted on the cancer mortality data of the elderly aged ≥60 years in China, which were selected from the Chinese Cancer Registry Annual Report. Joinpoint regression model was used to calculted average annual percentage change (AAPC) to estimate the time trends. Age-period-cohort (APC) model was used to explore the age, period and birth cohort effect on the risk of cancer death. Bayesian age-period-cohort (BAPC) model was used to predict trends in cancer mortality among elderly by gender and region to 2035.

**Results:**

2005–2016, cancer mortality in the elderly in China showed a decreasing trend (AAPC = -1.2%, *P*<0.001). Cancer mortality in rural areas was higher than that in urban areas, but the urban-rural difference gradually narrowed (*t* = 6.1, *P*<0.01). The APC model showed that cancer mortality increased with age. The relative risk (RR) for the period effect decreased. RR was lower for the later- born cohort than that for the earlier-born cohort in rural areas. Lung cancer mortality ranked first in both male and female, and showed an increasing trend among female in the 60–64, 80–84 and ≥85 age groups (AAPC_60-64_ = 1.0%, AAPC_80-84_ = 0.8%, AAPC_≥85_ = 2.0%, all *P*<0.05). By 2035, cancer mortality for the elderly was predicted to decline nationally, by sex and in rural areas, while rising in urban areas.

**Conclusion:**

Cancer mortality in the elderly in China showed a decreasing trend from 2005 to 2016, but it was still higher than the world average. Early cancer screening is important, especially in the elderly male and in rural areas.

## Introduction

A country or region entering an aging society is usually characterized by 10% or 7% of the total population being aged ≥60 or ≥65 years, respectively. Data from the seventh population census showed that the number of elderly aged ≥65 in China was risen from 88.21 million in 2000 to 190 million in 2020, with the proportion of the population in this age group being increased by 6.5% in the past 20 years [1]. This new demographic change indicates that the aging of the population in Chinese society has deepened.

Cancer has become one of the major problems that seriously threaten the life and health of Chinese residents with the emergence and aggravation of the aging society, as well as the changes of human living environment and behavior patterns. Because the growth of age will promote the atrophy and function decline of the body’s parenchymal organs, increasing the susceptibility and risk of cancer in the elderly [2–5]. Studies had shown that cancer mortality in the elderly in China from 2006 to 2010 was 786.53 per 100,000 adults, which was 13.53 times higher than that of the middle-aged and young adults (58.14 per 100,000 adults) [6]. Data released by the International Agency for Research on Cancer for 2021 showed that China had approximately 3 million cancer deaths, with the elderly accounting for approximately 72%, a mortality rate higher than the global average [7].

A comprehensive understanding of the current situation and long-term trend of cancer in the elderly population is of great significance for accurate prevention and control of cancer occurrence and development. In this study, the Joinpoint model and Bayesian age-period-cohort (BAPC) model were constructed to analyze the current situation and future trend of cancer death in the elderly in China from 2005 to 2035 by gender and region, and the age-period-cohort (APC) model was used to analyze the impact of age, period and cohort on mortality, so as to grasp the characteristics of cancer prevalence in the elderly in China, and to provide reference for formulating feasible cancer prevention and control measures.

## Materials and methods

### Data sources

Cancer mortality data for the elderly aged ≥60 years in China from 2005 to 2016 were selected from the Chinese Cancer Registry Annual Report from 2008 to 2019 published by the National Cancer Center [8–19].

### Index calculation

Mortality rates, age/sex/regional-specific mortality rates and standardized mortality rate (SMR) were calculated for all cancers (C00-C96) according to the International Classification of Diseases (ICD-10).

### Joinpoint regression analysis

Average annual percentage change (AAPC) was calculated using Joinpoint regression model to describe trends in cancer mortality in the elderly. Monte Carlo arrangement test is used to determine the number of connection points, the position of each connection point and its corresponding P-value, and the test level was α = 0.05(two-sided test). The Joinpoint regression model is divided into linear and log-linear model. The log-linear model is generally chosen when analyzing the trend of cancer mortality based on population [20]:

$$E[y|x]=e^{\beta_{0}+\beta_{1}^{x+}\delta_{1}{\left(x-\tau_{1}\right)}^{+}+\cdots +\delta_{k}(x-\tau_{k})}$$

Where *e* is the natural base, *k* indicates the number of turning points, *τ*_*k*_ indicates the unknown turning points, *β*_0_ is the invariant parameter, *β*_1_ is the regression coefficient, *δ*_*k*_ indicates the regression coefficient of the segment function in paragraph *k*. When (*x*−*τ*_*k*_)>0, ${\left(x-\tau_{1}\right)}^{+}=x-\tau_{k}$, otherwise ${\left(x-\tau_{1}\right)}^{+}=0$.

Calculation formula of APC: $APC=(e^{\beta 1}‐1)\times 100$

Calculation formula of AAPC: $\mathrm{AAPC}=[\exp(\sum w_{i}\beta_{i}/\sum w_{i})‐1]\times 100$ where *β*_1_ is the regression coefficient, *w*_*i*_ is the width of the interval span (i.e., the number of years included in the interval) for each segmentation function, and *β*_*i*_ is the regression coefficient corresponding to each interval.

If AAPC>0, it means that the mortality is increasing year by year, and vice versa, it means that the mortality is decreasing year by year.

### Age-period-cohort model

The APC model is based on Poisson distribution to estimate the risk of death in the population, reflecting trends in cancer by age, period and cohort, adjusting for age, period, and cohort [21]. In this study, data were aggregated to fit model conditions with the same age, period, and cohort interval prior to model analysis. Time, age and cohort were divided into 2 periods (2005–2010, 2010–2015), 6 groups (60–64, 65–69, 70–74, 75–79, 80–84, ≥85) and 8 birth cohorts (1920–1924,. . .,1955–1959) with intervals of 5 years, respectively. The APC model can be written as [22]:

$$\log(R)=\mu +\alpha Age_{i}+\beta Period_{j}+\gamma Cohort_{k}$$

Where *R* is the outcome variable for an event, such as mortality (×10^5^), *α*, *β* and *γ* represent the regression coefficients for the age, period, and cohort effects, respectively. *μ* represents the mean effect. The model parameters (*α*, *β*, *γ*) are exponentially transformed to represent the relative risk (RR) of a particular age, period and birth cohort relative to each mean level. The APC model was performed using the Stata 15.0 software.

### Bayesian age-period-cohort model

The Bayesian age-period-cohort (BAPC) model is based on the APC model, which describes trends in disease by considering the effects of age, period, and cohort factors on mortality rates. Using the APC model, it is possible to predict future mortality. However, because of the linear relationship between age, period, and cohort factors in the model, which leads to difficulty in parameter estimation, a Bayesian model is added to the APC model. It is able to estimate the a priori information of the unknown parameter and the sample information comprehensively to derive the posterior distribution, and infer the unknown parameter according to the posterior distribution, which is commonly used in integrated nested Laplace approximation algorithm for estimation to directly approximate the posterior marginal distribution, avoiding the mixing and convergence problems [23, 24], The formula equation is:

$$f\left(\alpha |k_{\alpha }\right)\propto k_{\alpha }^{\frac{I-2}{2}}\exp\left(-\frac{k_{\alpha }}{2}\sum_{i=3}^{I}{\left(\alpha_{i}-2\alpha_{i-1}+\alpha_{i-2}\right)}^{2}\right)=k_{\alpha }^{\frac{I-2}{2}}\exp\left(-\frac{1}{2}\alpha^{T}Q\alpha \right)$$

where *k* is the precision parameter; *Q* is the rank; and *α*_*i*_ *(i = 1*, *…*, *I)* is the age effect. The BAPC model was created using the BAPC and INLA package of R 4.3.1 software.

## Result

### Cancer mortality in the elderly in China, 2005–2016

#### Overall cancer mortality in the elderly in China

A total of 2,590,265 people aged ≥60 years died of cancer in China from 2005 to 2016, with a median crude mortality of 770.4 per 100,000 individuals (729.3/100,000–837.3/100,000), showing a decreasing trend over time (APC = -1.2%, *P*<0.001). Among them, cancer deaths in the group aged 60–64 years accounted for 17% (431,603 deaths), the groups aged 65–69, 70–74, 75–79, 80–84, and ≥85 years accounted for 17% (450,614 deaths), 19% (498,679 deaths), 21% (532,027 deaths), 16% (411,330 deaths), and 10% (266,012 deaths), respectively (**Table 1**).

**Table 1:** Mortality of cancer in the elderly in China from 2005 to 2016.

Year	Number of deaths	Mortality rate	SMR
		per 100000	per 100000
2005	65604	810.4	776.5
2006	74337	837.3	801.8
2007	75518	832.7	795.1
2008	87912	829.0	782.1
2009	110316	831.2	793.2
2010	158251	827.4	794.1
2011	185860	812.7	786.2
2012	248439	786.9	765.3
2013	291170	778.4	761.1
2014	367025	747.9	732.1
2015	420164	738.3	723.8
2016	505669	729.3	717.7
AAPC (%)	—	-1.2	-0.9
95%CI (%)	—	(-1.6, -0.8)	(-1.3, -0.4)
T	—	-5.9	-3.9
P	—	<0.001	<0.001

SMR, standardized mortality rate. AAPC, average annual percentage change. 95%CI, 95% confidence interval

#### Sex-specific cancer mortality in the elderly in China

**Fig 1:**
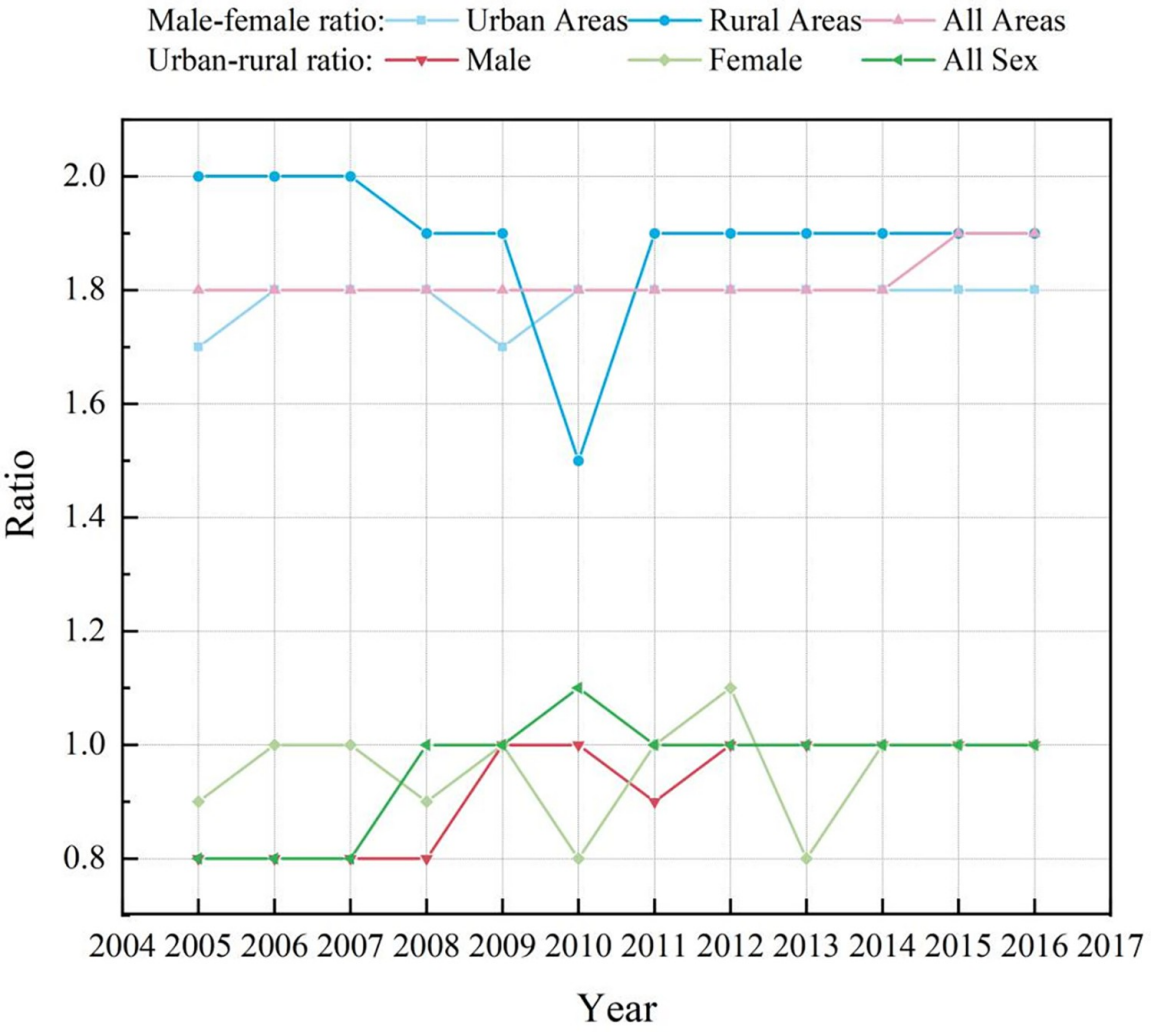
Urban-rural ratio and male-female ratio of cancer mortality in the elderly in China from 2005 to 2016.

A total of 63% (1,633,188 deaths) of the all cancer deaths occurred in male and 37% (9,570,77 deaths) in female between 2005 and 2016. Cancer mortality showed a decreasing trend in the older male and female, but the decreasing trend was more obvious in the latter (AAPC_male_ = -1.1%, AAPC_female_ = -1.4%, all *P<*0.05). In addition, from the perspective of different regions, cancer mortality in the older male and female in rural areas decreased significantly relative to that in urban areas (rural: AAPC_male_ = -3.1%; AAPC_female_ = -2.8%, all *P<*0.05; urban: AAPC_male_ = -0.6%, AAPC_female_ = -1.0%, all *P<*0.05). However, male in rural areas showed a faster decline, whereas female in urban areas showed a more significant decline, and the sex difference widened year by year (*t* = 2.7, *P<*0.05) (**Table 2 and Fig 1**).

**Table 2:** AAPC of cancer mortality in the elderly with different characteristics in China from 2005 to 2016 (%).

Age	Urban Areas			Rural Areas			All Areas		
	Male	Female	Both	Male	Female	Both	Male	Female	Both
60–64	1.3^a^	0.5	1.1^a^	-3.9^a^	-4.0^a^	-4.0^a^	0.0	-0.4	0.0
65–69	0.8^a^	-1.3^a^	0.1	-2.5^a^	-2.3^a^	-2.4^a^	0.3	-1.1^a^	-0.1
60–69	0.7^a^	-0.9^a^	0.2	-3.3^a^	-3.3^a^	-3.3^a^	-0.1	-1.1^a^	-0.3^a^
70–74	1.0^a^	-2.0^a^	-1.3^a^	-3.6^a^	-2.9^a^	-3.1^a^	-1.3^a^	-2.0^a^	-1.6^a^
75–79	1.6^a^	-1.3^a^	-1.2^a^	-3.2^a^	-1.1^a^	-2.5^a^	-1.8^a^	-1.6^a^	-1.7^a^
70–79	-1.0^a^	-1.3^a^	-1.1^a^	-3.1^a^	-3.0^a^	-3.1^a^	-1.4^a^	-1.7^a^	-1.5^a^
80–84	-1.2^a^	-0.2	-0.6	-2.1^a^	-1.1^a^	-1.2^a^	-1.5^a^	-0.6^a^	-1.0^a^
85+	-0.6	0.7	0.3	0.2	0.2	0.5	-1.0	0.0	-0.3
All	-0.6^a^	-1.0^a^	-0.7^a^	-3.1^a^	-2.8^a^	-2.8^a^	-1.1^a^	-1.4^a^	-1.2^a^

AAPC, average annual percentage change. a, *P*<0.05

#### Regional-specific cancer mortality in the elderly in China

A total of 57% (1,483,037 deaths) and 43% (1,107,228 deaths) of cancer deaths among the older individuals occurred in urban and rural areas, respectively, from 2005 to 2016. Over time, cancer mortality decreased in both urban and rural areas, but it decreased more significantly in rural areas (urban: AAPC = -0.7%; rural: AAPC = -2.8%, all *P*<0.05). In addition, the ratio of mortality between urban and rural areas also increased with time, gradually approaching 1, and the difference between urban and rural areas decreased (*t* = 4.3, *P*<0.01). The urban-rural mortality difference was also narrowed by sex, especially for male (*t* = 6.1, *P*<0.01) (**Table 2 and Fig 1**).

#### Type-specific cancer mortality in the elderly in China

**Fig 2:**
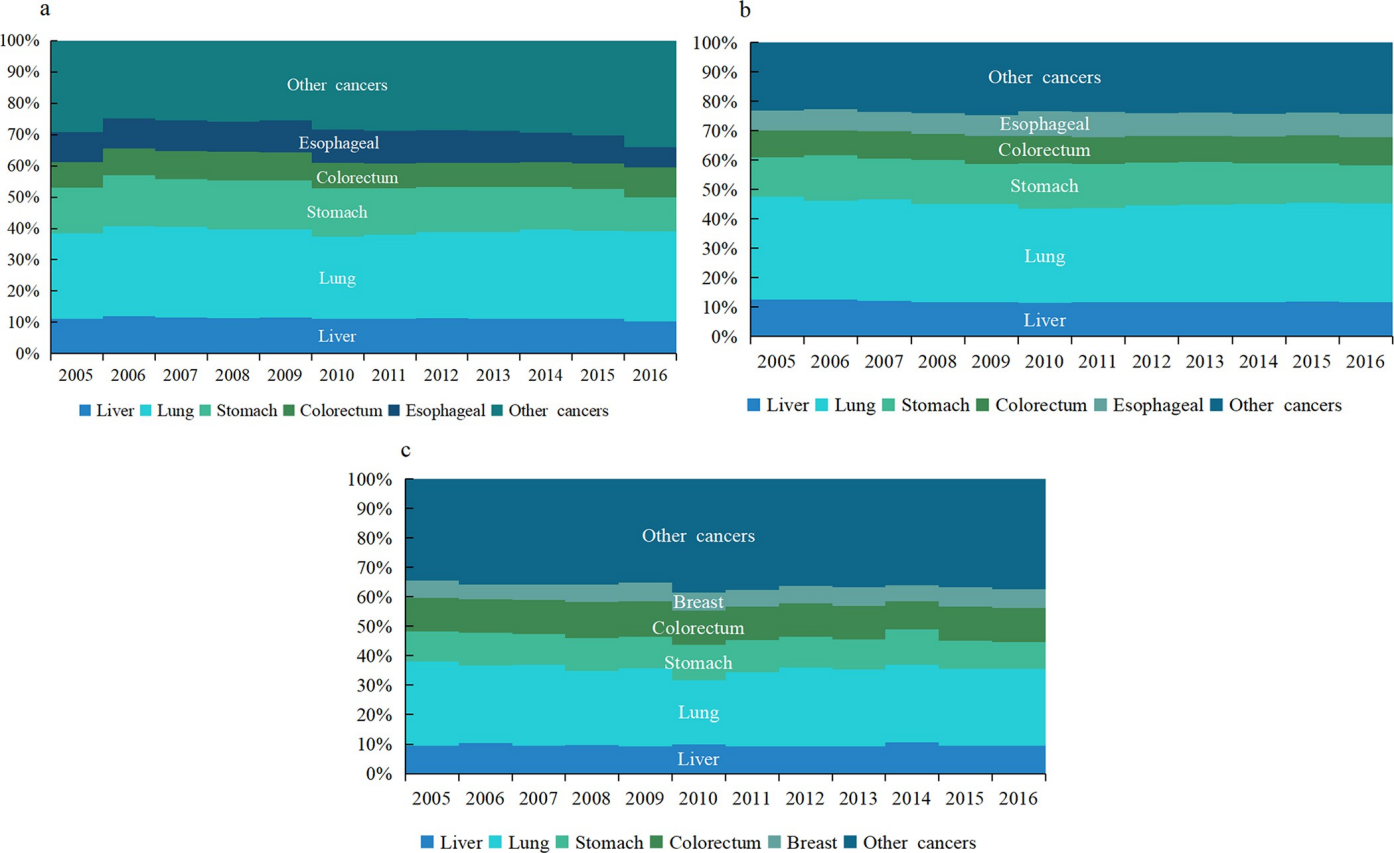
Composition of cancers in the elderly in China from 2005 to 2016. (a: all; b: male; c: female).

Lung cancer ranked first in the number of cancer-related deaths in the elderly in China from 2005 to 2016, with approximately 743,922 deaths. As for the order of cancer deaths, liver, stomach, esophageal and colorectal cancers followed, and the top five cancer deaths accounted for about 73.0% of all cancer deaths (**Fig 2A**). In male, lung cancer ranked first, with 506,578 deaths, followed by gastric, liver, esophageal and colorectal cancers. The top five cancers accounted for 77.0% of all cancer deaths (**Fig 2B**), with lung cancer ranking first in female, with about 237,340 deaths, followed by colorectal, stomach, liver, and breast cancers. The top five cancers accounted for 62.0% of all cancer-related deaths (**Fig 2C**).

**Fig 3:**
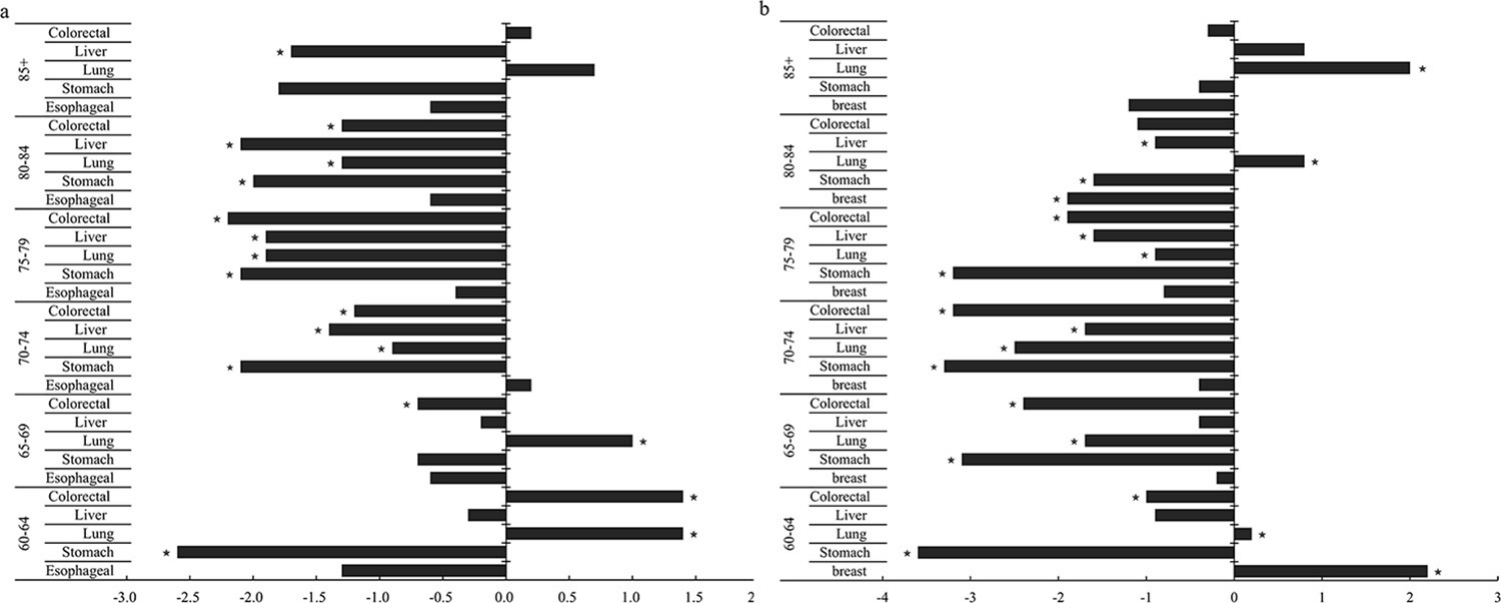
Mortality trend of the top five cancers among the elderly of different genders and age groups in China from 2005 to 2016. (a: male; b: female). ★, *P*<0.05.

For older male, lung cancer mortality ranked first in all age groups in China from 2005 to 2016. The lung cancer mortality in the groups aged 60–64 and 65–69 years showed an increasing trend (AAPC_60-64_ = 1.4%, AAPC_65-69_ = 1.0%, all *P*<0.05), while that in the groups aged 70–74, 75–79, 80–84 and ≥85 years showed a decreasing trend (AAPC_70-74_ = -0.9%, AAPC_75-79_ = -1.9%, AAPC_80-84_ = -1.3%, all *P*<0.05; AAPC_≥85_ = -0.7%, *P*>0.05). Liver cancer mortality showed an obscure change in male aged 60–64 and 65–69 years, while a decreasing trend was observed in the groups aged 70–74, 75–79, 80–84 and ≥85 years (AAPC_70-74_ = -1.4%, AAPC_75-79_ = -1.9%, AAPC_80-84_ = -2.1%, AAPC_≥85_ = -1.7%, all *P*<0.05). Colorectal cancer mortality presented an increasing trend in male aged 60–64 and 65–69 years (AAPC_60-64_ = 1.4%, AAPC_65-69_ = 0.7%, all *P*<0.05), while it decreased with increasing age in the group aged 70–74, 75–79, 80–84 and ≥85 years. Esophageal cancer mortality was stable in all male age groups (*P*>0.05). Moreover, stomach cancer mortality in all male age groups showed a decreasing trend, and the decrease rate in the group aged 60–64 years was more obvious than in other age groups (AAPC_60-64_ = -2.6%, *P*<0.05) (**Fig 3A**).

Lung cancer mortality in older female showed an increasing trend in the groups aged 60–64, 80–84, and ≥85 years (AAPC_60-64_ = 1.0%, AAPC_80-84_ = 0.8%, AAPC_≥85_ = 2.0%, all *P*<0.05). Liver cancer mortality was relatively stable in the group aged ≥85 years (APC_≥85_ = 0.8%, *P*>0.05), while the other age groups showed a decreasing trend (AAPC_60-64_ = -0.9%, AAPC_65-69_ = -0.4%, all *P*>0.05; AAPC_70-74_ = -1.7%, AAPC_75-79_ = -1.6%, AAPC_80-84_ = -0.9%, all *P*<0.05). Colorectal cancer in female of all age groups showed a downward trend. In addition, similar to male, stomach cancer mortality showed a decreasing trend in all age groups, and the decrease rate was more obvious in the group aged 60–64 years than the other age groups. However, the breast cancer mortality in the group aged 60–64 years showed an increasing trend (AAPC_60-64_ = 2.2%, *P*<0.05), while that in the group aged 80–84 years showed a decreasing trend (AAPC_80-84_ = -1.9%, *P*<0.05), and the rest of the group presented a steady trend (AAPC_65-69_ = -0.2%,AAPC_70-74_ = -0.4%,AAPC_75-79_ = -0.8%, APC_≥85_ = -1.9%, all *P*>0.05) (**Fig 3B**).

### Age-period-cohort analysis

#### Age effect

Among Chinese elderly aged 60 years and older, cancer mortality tends to increase for male, female, and in rural areas, then decreases with age and peaks in the 80–40 age group, while in urban areas, cancer mortality all increases with age.

#### Period effect

For both male and female, RR remained stable from 2005 to 2010 and showed a decreasing trend from 2010 to 2015. From 2005 to 2010, RR in urban areas showed an increasing trend from 2005 to 2010 and a decreasing trend from 2010 to 2015; while RR in rural areas decreased from 2005 to 2015 in both.

#### Cohort effect

**Fig 4:**
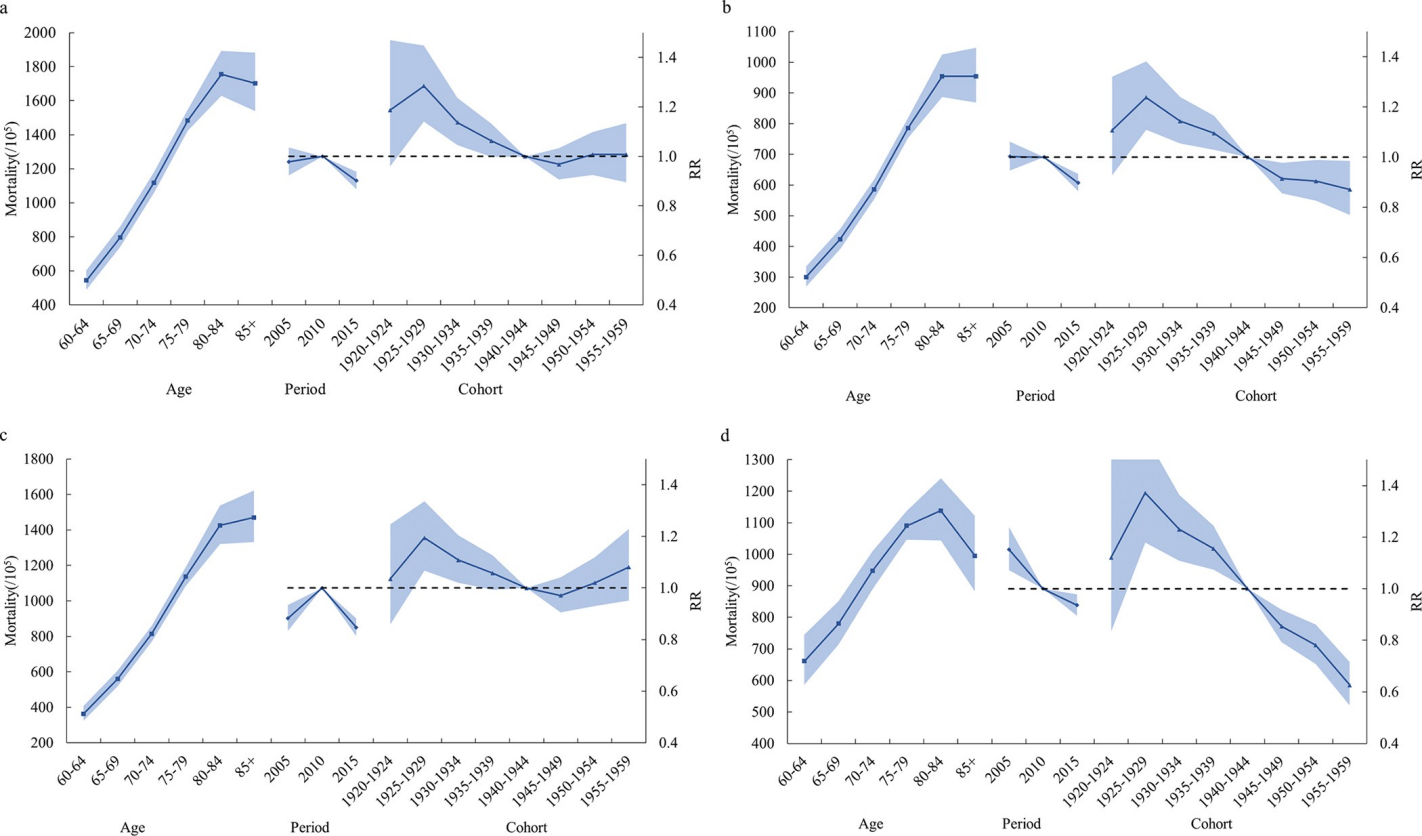
Age-period-cohort model of cancer mortality in Chinese elderly aged 60 and above from2005 to 2016. (a: male; b: female; c: urban; d: rural). RR, Risk Ratio.

For both male and female, urban and rural, RR declined for birth cohorts born between 1925 and 1949, whereas for birth cohorts born between 1950 and 1959, RR continued to decline for female and rural areas, while it stabilized for male and increased for urban areas (**Fig 4**).

### Prediction of cancer mortality in the elderly in China, 2017–2035

As predicted by the BAPC model, the cancer mortality in the elderly in China showed a continuous downward trend during 2017–2035, with the mortality rate in 2017, 2020, 2025, 2030 and 2035 being 684.21/100,000 (95%CI: 683.75, 684.67), 663.61/100,000 (95% CI: 663.16, 664.06), 638.17/100,000 (95%CI: 637.72, 638.61), 619.63/100,000 (95%CI: 619.19, 620.07) and 604.21/100,000 (95%CI: 603.45, 604.32), respectively. Trends in mortality rates for males, females, and rural areas were similar to those of the country as a whole, while that in urban areas showed an increasing trend.

**Fig 5:**
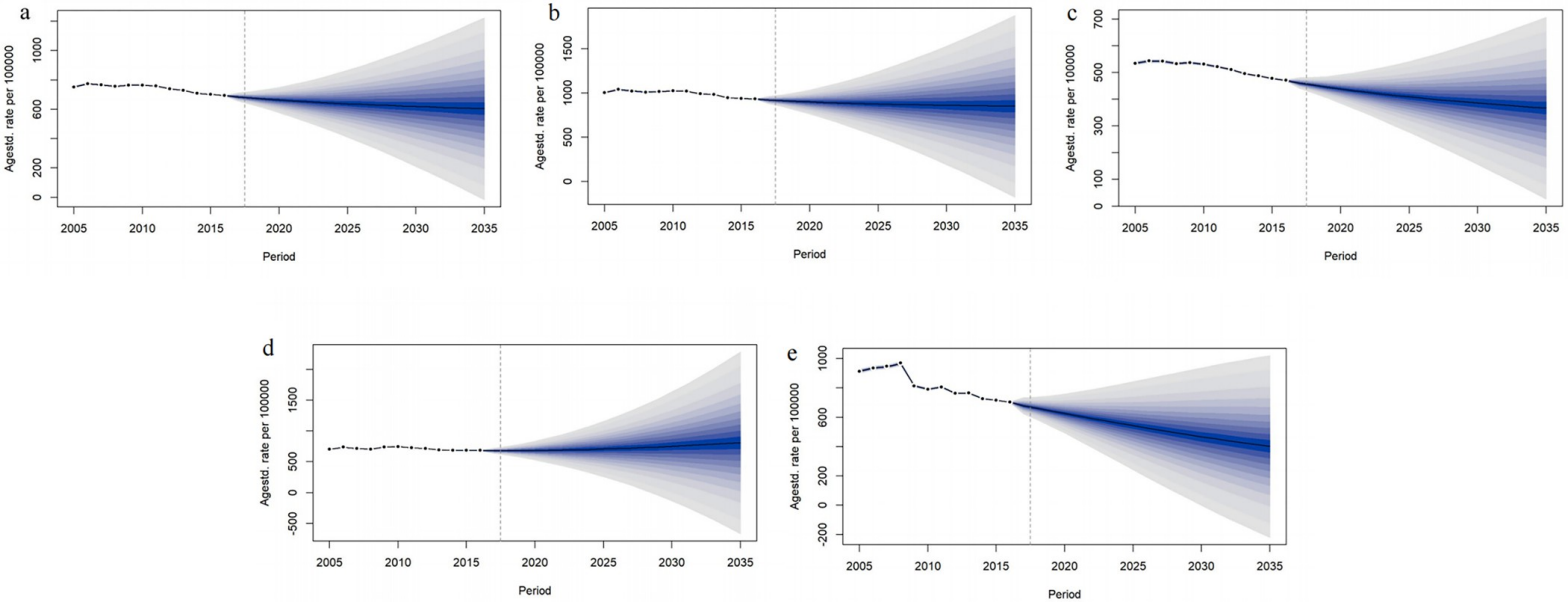
Cancer mortality in Chinese elderly from 2017 to 2035. (a: all; b: male; c: female; d: urban; e: rural).

The mortality for male was predicted to remain greater than that for female, declining from 921.69/100,000 (95%CI: 921.16, 922.22) and 461.09/100,000 (95%CI: 460.71, 461.47) in 2017 to 850.77/100,000 (95%CI: 850.27, 851.28) and 367.20/100,000 (95%CI: 366.85, 367.54), respectively. The mortality rates for urban and rural areas changed from 679.75/100,000 (95%CI: 679.30, 680.21) and 678.53/100,000 (95%CI: 678.07, 678.99) in 2017 to 809.46/100,000 (95%CI: 808.97, 809.96) and 400.64/100,000 (95%CI: 400.29, 401.00) in 2035, respectively (**Table 3 and Fig 5**).

**Table 3:** Cancer mortality in the elderly in China, 2017–2035 (/100,000).

Year	National		Male		Female		Urban		Rural	
	SMR	95% CI	SMR	95% CI	SMR	95% CI	SMR	95% CI	SMR	95% CI
2017	684.21	683.75, 684.67	921.69	921.16, 922.22	461.09	460.71, 461.47	679.75	679.30, 680.21	678.53	678.07, 678.99
2018	676.63	676.17, 677.08	913.57	913.05, 914.10	453.17	452.79, 453.55	679.79	679.33, 680.25	660.93	660.48, 661.39
2019	669.74	669.29, 670.20	906.30	905.78, 906.82	445.80	445.42, 446.17	681.08	680.62, 681.54	643.11	642.66, 643.55
2020	663.61	663.16, 664.06	900.08	899.56, 900.60	438.92	438.54, 439.29	683.40	682.94, 683.86	625.75	625.31, 626.19
2021	657.85	657.40, 658.31	894.38	893.86, 894.90	432.39	432.02, 432.76	686.49	686.03, 686.95	608.87	608.43, 609.30
2022	652.28	651.83, 652.73	888.99	888.47, 889.51	426.11	425.74, 426.48	690.34	689.88, 690.81	592.02	591.59, 592.45
2023	647.10	646.66, 647.55	884.20	883.68, 884.72	420.15	419.79, 420.52	695.28	694.81, 695.74	575.29	574.86, 575.71
2024	642.39	641.95, 642.84	879.97	879.45, 880.48	414.60	414.24, 414.97	701.24	700.78, 701.71	558.74	558.32, 559.15
2025	638.17	637.72, 638.61	876.38	875.87, 876.90	409.40	409.03, 409.76	708.09	707.62, 708.55	542.69	542.28, 543.10
2026	634.14	633.70, 634.58	873.04	872.52, 873.55	404.44	404.08, 404.8	715.56	715.09, 716.03	527.09	526.69, 527.50
2027	630.20	629.75, 630.64	869.81	869.29, 870.32	399.65	399.29, 400.00	723.63	723.16, 724.10	511.72	511.32, 512.12
2028	626.45	626.01, 626.89	866.86	866.35, 867.37	395.07	394.72, 395.42	732.47	732.00, 732.95	496.63	496.23, 497.03
2029	622.92	622.48, 623.36	864.19	863.68, 864.71	390.70	390.35, 391.05	742.02	741.54, 742.50	481.85	481.46, 482.24
2030	619.63	619.19, 620.07	861.84	861.32, 862.35	386.52	386.17, 386.88	752.18	751.70, 752.66	467.50	467.12, 467.89
2031	616.40	615.97, 616.84	859.54	859.03, 860.05	382.49	382.14, 382.84	762.79	762.30, 763.27	453.53	453.15, 453.91
2032	613.19	612.75, 613.62	857.23	856.72, 857.74	378.54	378.19, 378.88	773.78	773.29, 774.26	439.84	439.47, 440.22
2033	610.02	609.58, 610.46	854.99	854.48, 855.50	374.68	374.33, 375.03	785.24	784.75, 785.73	426.45	426.08, 426.82
2034	606.92	606.49, 607.36	852.83	852.32, 853.34	370.91	370.56, 371.25	797.16	796.67, 797.65	413.37	413.01, 413.73
2035	604.21	603.45, 604.32	850.77	850.27, 851.28	367.20	366.85, 367.54	809.46	808.97, 809.96	400.64	400.29, 401.00

Note: SMR, standardized mortality rate; CI, confidence interval

## Discussion

It had been reported that the global death from cancer in 2020 was nearly 10 million, with nearly 3 million deaths being reported in China, accounting for 30.2% [25]. Therefore, the National Health Commission and other departments jointly formulated the Healthy China Initiative (2019–2030), which aim to “significantly reduce the premature mortality caused by major chronic diseases and greatly improve the average healthy life expectancy” and regarded the prevention and control of cancer in the elderly as a priority. Based on the cancer mortality data in the elderly from 2005 to 2016 collected by the National Cancer Registry Center, the cancer mortality in the elderly in China showed a decreasing trend during this period, especially in rural areas, male, and the elderly aged >80 years, but they were still higher than those in the global, urban areas, female, and the younger adults, respectively. Lung, liver, and colorectal cancers were the three main types of cancer affecting the health of the elderly in China. Cancer mortality for the elderly was predicted to show a decreasing trend nationally, by gender and rural areas from 2017 to 2035, except for an increasing trend in urban areas.

Cancer mortality in the elderly in China showed a decreasing trend from 2005 to 2016, indicating that cancer prevention and treatment in China had effectively reduced the harm caused by cancer to some extent. Data released by the International Agency for Research on Cancer in 2021 showed that the mortality rate of cancer among the elderly in China was 3.2 times that in the United States [7]. Furthermore, cancer has caused a severe loss of healthy living in China. Data showed that the disability-adjusted life caused by various cancers accounted for 15.3% of the total disability-adjusted life in China in 2017, which was almost twice the global average [26]. This indicates that the overall diagnosis and treatment rates of patients with cancer in China may not be high. For example, 90% of stomach cancers in China were in the progressive stage, and the diagnosis and treatment rate were <10%, which was much lower than that in Japan (70%) and Korea (50%) [27]. Therefore, early detection and diagnosis of cancer are key to treating cancer and prolonging the survival rate.

Cancer mortality in elderly male in China from 2005 to 2016 was higher than that in female. This was in agreement with previous research [28], possibly because male have more chances to be exposed to cancer risk factors in daily life. Moreover, cancer mortality in the elderly in rural areas of China was generally higher than that in urban areas from 2005 to 2016. However, cancer mortality in rural areas showed a declining trend, whereas that in urban areas was more stable. The urban and rural cancer differences were narrowing, possibly because of the narrowing of risk factors for cancer, such as smoking, drinking, chronic infection, dietary habits, and indoor and outdoor air pollution. As a result, cancer incidence in urban and rural areas had become increasingly similar [29]. Owing to the lack of medical resources in rural areas and the weak awareness of cancer prevention, cancer mortality is still high in rural areas. Therefore, it is suggested that corresponding cancer prevention and control measures should be developed in urban and rural areas by combining the characteristics of death prevalence.

The age effect in the age-period-cohort model was mainly embodied by the risk of death due to age and the impact of changes in physiological states on individual growth [30]. The age effect on cancer mortality risk in the elderly in this study increased with age, which was consistent with the results of Zhong et al. [21]. However, the declining trend in cancer mortality among the elderly after 85 years of age was due to the increase in basic diseases and competitive death from other diseases [31]. Male have a higher chance of accumulating exposure to risk factors of cancer than female, results in a higher overall mortality rate for male than for female. Among those younger than 85 years of age, the trend of cancer mortality changes more slowly in rural than in urban areas and decreases significantly after the age of 85. This change has brought severe challenges to the allocation of medical resources in China, especially resources for cancer prevention, diagnosis, treatment, and prognosis in the elderly. Therefore, cancer prevention and treatment in the elderly should not only focus on clinical diagnosis and treatment, but also conduct health education on cancer prevention and control of common risk factors in the elderly to expand the focus of medical work from disease treatment to lifestyle prevention [32].

Cohort effects can be viewed as the interaction of increasing age and period changes. As evidenced by the fact that changes in exposure risk factors following exposure to a specific historical event do not affect all age groups simultaneously, and different birth cohorts ten to have different risk profiles [33]. The study found that the cohort effect of mortality in both male and female, urban and rural areas showed acontinuous decline from 1925–1949, with the birth cohort showing a faster decline in RR for female and rural areas and a gradual stabilization thereafter for male. One reason for this is that more people in the later cohort were more aware of health and disease prevention and better educated than in the earlier cohort, thereby reducing the risk of cancer death occurrence [34]. The cohort effect of mortality in urban areas showed an increasing trend between 1950–1959.This is consistent with the findings of Jiang et al. [35]. Which suggest that access to health care may be weaker in rural areas of China than in urban areas, and that differences in socioeconomic status contribute to differences in cancer incidence and subsequent cancer mortality [36].

According to the current project research results, lung, liver, stomach, esophageal, and colorectal cancers ranked in the top five in terms of death composition of cancers in the elderly in China, accounting for 73.0%. These findings were consistent with those of Zhang et al. [37], suggesting that key monitoring is required in the future. Among these, lung cancer was the leading cause of death in both male and female. There are many studies on the risk factors of lung cancer, with smoking being recognized as the main one. Wen et al. [38] reported a significant increase in lung cancer mortality among non-smoking Chinese female exposed to tobacco smoke at work. In addition, Gao et al. [39] found that the risk of lung cancer in people exposed to secondhand smoke was 1.31 times higher than that in people not exposed to secondhand smoke. Therefore, establishing effective national anti-smoking laws in China can help significantly reduce smoking in public places. The mortality rates of both stomach and liver cancers in China showed an overall decreasing trend between 2005 and 2016, which was consistent with the results of other domestic studies [40, 41]. Colorectal cancer mortality was on the rise in the age group of 60–69 years old in male, which was consistent with the results of domestic studies [42]. However, compared with the decreasing trend of colorectal cancer mortality in developed countries, represented by the United States year by year [43], China should improve residents’ awareness of cancer prevention and actively carry out early screening for colorectal cancer. The mortality rate of breast cancer showed a steady trend. However, among the top five cancer-related deaths among female, breast cancer is still a dominant cancer that affects the life and health of female residents in China. The results of the current project indicated that the mortality of breast cancer in older Chinese female aged 60–64 years still presents a rising trend from 2005 to 2016. In the face of this severe situation, China should pay close attention to the incidence of breast cancer in female of all ages and improve the physical examination system.

This study has some limitations. The data used in this study were obtained from the Chinese Cancer Registry Database, which collects information on cancer cases through systematic collection, collation, preservation, statistics, and analyses. However, considering coverage, data completeness may cause some bias in the results. In addition, it is difficult to avoid biological fallacies when conclusions obtained from fitting the age-period-cohort model are extrapolated to individuals. Therefore, the hypothesis presented in this paper only provides clues to the etiology and needs to be confirmed by further epidemiological analyses in the future.

In summary, the overall cancer mortality in the elderly in China shows a downward trend, but is still higher than the global average. In addition, as for the death characteristics of elderly patients with cancer in China, there are still differences in age, sex, urban and rural areas, and cancer types. It is suggested to timely adjust prevention and control priorities in China in the future, consider the elderly population in urban and rural areas, optimize the prevention and control sequence, rationally allocate medical resources, conduct targeted prevention and control of specific tumors based on different sexes and ages, improve the early detection rate of cancers in order to enhance the quality of life of patients, and prolong the survival time of patients.
